# Anti-diabetic potential of *Artemisia monosperma* Delile extract related to anti-inflammatory, antioxidant, and wound-healing properties

**DOI:** 10.1038/s41598-026-50561-8

**Published:** 2026-05-06

**Authors:** Hanaa B. Atya, Mohamed S. Mady, Ola Nosseir, Fatma A. Moharram, Nashwa F. Tawfik, Nashwa E. Hashad

**Affiliations:** 1 Department of Biochemistry and Molecular Biology, Faculty of Pharmacy, Capital University (formerly Helwan University), Cairo, 11795 Egypt; 2 Department of Pharmacognosy, Faculty of Pharmacy, Capital University (Formerly Helwan University), Cairo, 11795 Egypt; 3 Department of Pharmaceutical Chemistry, Faculty of Pharmacy, Capital University (formerly Helwan University), Cairo, 11795 Egypt

**Keywords:** Antidiabetic, Anti-inflammatory, Antioxidant, *Artemisia monosperma*, Polyphenols, Docking study, Biochemistry, Computational biology and bioinformatics, Drug discovery, Plant sciences

## Abstract

*Artemisia monosperma* Delile (Asteraceae) is traditionally used for gastrointestinal issues and diabetes. This study aimed to characterize its phenolic profile and evaluate the molecular basis for its antioxidant, anti-inflammatory, and antidiabetic properties. The defatted 80% aqueous methanol extract (DAME) of A. monosperma aerial parts was profiled by HPLC-HRMS to identify phenolic compounds. Antihyperglycemic activity was tested via the Sucrose Loading Model (SLM) and a Type 2 diabetes model. Anti-inflammatory effects were measured in LPS-stimulated RAW264.7 cells by TNF-α and CRP gene expression. Wound-healing potential was evaluated in BJ cells, and antioxidant activity was assessed against superoxide, DPPH, and NO radicals. Molecular docking was used to examine the binding of the identified metabolites to targets, including inducible nitric oxide synthase (iNOS) and sulfonylurea receptor 1 (SUR1). The HRHPLC/MS analysis of the DAME of A. monosperma tentatively identified 25 secondary metabolites in the negative mode, including twelve phenolic acid derivatives, eight flavonoid compounds, and five miscellaneous terpenes. The DAME exhibits potent antihyperglycemic activity, with a 300 mg% dose reducing blood glucose levels by approximately 26% in a Type 2 diabetes model, comparable to diamicron. It also shows strong anti-inflammatory effects, reducing TNF-α and CRP gene expression by 66% and 82%, respectively, in LPS-stimulated RAW264.7 cells. Furthermore, it enhances wound healing, achieving approximately 79% closure in treated BJ cells compared to 58% in controls, and exhibits antioxidant activity. The identified library of compounds **1**–**25** was virtually screened against (iNOS) and (SUR1) to assess their binding affinities for each target. The binding modes of the compounds with the lowest energy scores for each were investigated. Different compounds demonstrated stronger affinities to each target than the co-crystallized ligand, among which compounds **4**, **9**, **11**, and **14** possessed the lowest binding energies to both. *A. monosperma* DAME contains a variety of phenolic compounds and shows a potent multi-functional agent possessing anti-diabetic, anti-inflammatory, wound healing, and antioxidant activities, suggesting its therapeutic potential. However, more research is necessary to confirm its safety for clinical application.

## Introduction

Recently, exposure to different risk issues and harmful lifestyles has contributed to the progression of chronic degenerative diseases, such as diabetes mellitus^1^. Diabetes is a serious illness and is one of the top ten causes of death among adults worldwide^2^. It results from several disorders affecting fat, carbohydrate, and protein metabolism, caused by insulin insufficiency, hyperglycemia, or both, leading to metabolic alterations that can ultimately be fatal^3^. Diabetes is classified into three types: Type 1 diabetes mellitus (T1DM) is an autoimmune disease marked by insulin deficiency resulting from the loss of pancreatic β-cell function^4^. In contrast, type 2 diabetes mellitus (T2DM), a metabolic disorder, accounts for approximately 90% of cases and is characterized by hyperglycemia, primarily due to decreased insulin sensitivity in the liver, skeletal muscles, and adipose tissue, or impaired insulin signaling^2^. Gestational diabetes, which occurs during pregnancy and results from progressive insulin resistance, serves as a risk factor for developing T2DM later in life^4^. Chronic hyperglycemia caused by diabetes can lead to numerous functional and structural disorders because of the highest oxidative stress and inflammation^5^. Additionally, chronic diabetes can result in myocardial infarction and peripheral vascular disease, retinopathy, nephropathy, and neuropathy^6^. Inflammation is a self-defense mechanism to begin the healing process. Plentiful diseases, comprising diabetes, atherosclerosis, neurodegeneration, arthritis, Alzheimer’s disease, and cancer, are induced by chronic inflammation. Diabetes is a lifestyle ailment with persistent hyperglycemia and complications, including delayed wound healing, hypertension, neuropathy, and tissue hypoxia caused by impaired metabolism of carbohydrates, proteins, and lipids, leading to hyperglycemia^7^. Therefore, research into anti-inflammatory mechanisms may offer a novel approach to reducing the inflammatory consequences of diabetes and promoting wound healing. Despite recent advances in wound care products, traditional remedies based on natural ingredients, such as plant extracts and honey, remain promising alternatives. Moreover, achieving effective glycemic control is the primary goal of diabetes treatment, as it helps prevent microvascular, macrovascular, and neurological complications^8^. Excessive generation of reactive oxygen species (ROS) by hyperglycemia leads to oxidative damage to biomolecules, contributing to diabetic complications^9^. Excess free radicals deplete antioxidant defenses, thereby increasing oxidative stress in diabetes^10^. ROS-mediated tissue oxidation promotes insulin resistance, impairs insulin signaling, and causes β-cell destruction^11^. Moreover, it is linked to diabetes complications; consequently, reducing oxidative stress in diabetes can help minimize the damage to organs and enhance patients’ quality of life. Also, elevated oxidative stress often triggers chronic inflammation^12^, and the progression of diabetes is associated with heightened release of pro-inflammatory markers in response to oxidative stress. Excessive activation of the inflammatory response results in more organ damage, higher free radical levels, and the progression of diabetic complications. Consequently, anti-inflammatory agents play a crucial role in the treatment of diabetes.

Currently, diabetes management relies on anti-hyperglycemic drugs and insulin therapy. On the other hand, these treatments are not fully effective and may cause a range of side effects^13^. Moreover, they are often costly and difficult to access in developing countries^14^.

As a result, medicinal plants, particularly those rich in phenolic compounds, offer a promising source for developing new antidiabetic agents because of their effectiveness, accessibility, and low toxicity. In recent years, phenolic compounds have received significant attention in scientific research due to their antioxidant^13^, anti-inflammatory^15^, and antihyperglycemic activities^16^, making them promising agents for the management of diabetes and its complications^15^.

Genus Artemisia (Family Asteraceae) belongs to the tribe Anthemideae. It includes about 500 species of small shrubs and herbs worldwide, most of which are aromatic^16^. *Artemisia monosperma* Delile is commonly known as Sweet Annie, wormwood, or Qing Hao, and as Al-Ader’ in Egypt^17^. It is an aromatic, perennial green shrub native to Egypt and widely cultivated in other Arab countries, Africa, and China^18^. It is traditionally used, particularly by Bedouins of the Sinai Peninsula, for gastrointestinal disorders^19^, diabetes^20^, rheumatic pain, and fever^21^. Its dried leaves are also widely used in Egypt, alongside other Artemisia species, to prepare herbal tea with anthelmintic properties^22^. *A*. *monosperma* has been reported to be a rich source of various phenolic compounds^17,23–28^. Furthermore, it has been reported that the leaves of *A*. *monosperma* exhibit antimicrobial^29,30^, insecticidal^31,32^, and antimalarial^31,32^ properties. However, a scientific gap exists in understanding the mechanistic basis for these effects. Modern biological research establishes that chronic metabolic and digestive disorders are intrinsically linked to oxidative stress and sustained inflammatory responses^33^. Therefore, evaluating the antioxidant and anti-inflammatory activities of the DAME extract is necessary to validate its systemic therapeutic efficacy. Furthermore, since traditional skin treatments with *Artemisia* involve the repair of damaged tissue, investigating its wound-healing potential, which requires a coordinated reduction in inflammation and oxidative damage, offers a functional model to assess its regenerative properties^34^.

Additionally, continuation of our work on the aerial parts of *A. monosperma*^28^. This study aimed to tentatively identify the phenolic compounds in the defatted aqueous methanol extract (DAME) using HRHPLC/MS. Furthermore, it evaluates the antidiabetic, anti-inflammatory, antioxidant, and wound healing activities of DAME using in vivo and in vitro models to provide mechanistic validation of its traditional applications. Finally, to better understand the molecular basis of these activities, a comprehensive molecular docking analysis was performed to assess the binding affinities of the identified metabolites for key therapeutic targets, including inducible nitric oxide synthase (iNOS) and sulfonylurea receptor 1 (SUR1).

## Materials and methods

### Plant material

*A*. *monosperma* Delile aerial parts were collected from Al-Ein Alsokhna, a coastal region on the Red Sea in Egypt, in August 2022 at the end of the flowering stage, following the local garden`s guidelines for collection and complies with the collection legislation of Egypt, which comply with the IUCN policy statement on research involving species at risk of extinction and convention on the trade in endangered species of wild fauna and flora. Prof. Abduo M. Hamed, a Plant Ecology Professor at the Faculty of Science, Al-Azhar University, Cairo, Egypt, confirmed the plant identification. The plant was stored in the Pharmacognosy Department herbarium at the Faculty of Pharmacy, Capital University (formerly Helwan University), Cairo, Egypt, under the identifier 37 Amo 2/2023.

### Chemicals and reagents

Methanol, *n-*butanol, acetic acid, and *n*-hexane were supplied by El Nasr Pharmaceutical Chemicals Co. (Gesr El Suez, Cairo, Egypt). Streptozotocin, Naturstoff reagent, FeCl_3_, and ascorbic acid were purchased from Sigma-Aldrich (St. Louis, MO, USA). Diamicron→ 80 mg (Servier Egypt, Cairo Governorate, Egypt).

### Preparation of the defatted aqueous extract (DAME)

*A. Monosperma* aerial parts (250 g) were extracted with 80% aqueous methanol under reflux (3 × 4 L), then filtered. The solvents were removed under reduced pressure at 50 °C, yielding 15 g of dry extract. The residue was defatted with *n*-hexane under reflux (4 × 1.0 L), then the hexane was removed under reduced pressure at low temperature. The dried DAME (9 g) was subjected to 2-D paper chromatography (2D-PC) using the upper layer of *n*-butanol: acetic acid: water (4:1:5, BAW) for the first run, and 15% aqueous acetic acid (HAc), followed by examination under UV light before and after spraying with Naturstoff reagent and FeCl_3_ reagents.

### HPLC/HR MS analysis of DAME

The analysis utilized a 6530 Q-TOF LC/MS system (Agilent Technologies), equipped with an autosampler (G7129A), a Quat pump (G7104C), and a Column Comp (G7116A) for chromatographic separation. The injection volume was one µL. Analytes were separated on a Zorbax RP-18 column (Agilent Technologies; 150 mm × 3 mm, 2.7 μm) at a flow rate of 3 mL/min. Mass spectra were acquired in negative-ion mode using ESI, with a capillary voltage of 4000 V. Spectra ranged from m/z 50 to 3000. The gas temperature was maintained at 250 °C, and the drying-gas flow rate was 8 L/min. The skimmer and fragmentation voltages were set to 65 V and 130 V, respectively, and the collision energy was 10 V. The nebulization pressure was 58 psi.

Compound identification was preliminarily assigned based on accurate mass values from HPLC–HRMS analysis, along with comparisons with reported compounds in the literature and spectral databases. Because of the experimental setup used, MS/MS fragmentation data and Δppm mass accuracy values were unavailable; thus, the suggested compounds should be regarded as tentative annotations. Mass accuracy (Δppm) was determined by (measured m/z − theoretical m/z)/theoretical m/z × 10⁶. For some peaks, the Δppm values were relatively high, so the related compound identifications are considered tentative.

### Evaluation of antidiabetic activity

#### Animals

Male Wistar albino rats (200–250 g) obtained from VACSERA (Helwan, Cairo, Egypt) were housed at the Faculty of Pharmacy, Capital University (formerly Helwan University), animal facility, and provided with pelleted food and water. The rats were acclimatized to a controlled environment with a 12-hour light-dark cycle for one week. The study protocol was approved by the Faculty of Pharmacy, Capital University (formerly Helwan University), animal ethics committee (No. 14A2024), and was conducted in accordance with EC Directive 86/609/EEC for animal experiments.

At the end of the experimental period, rats were deeply anesthetized with an intraperitoneal injection of sodium pentobarbital (60 mg/kg). Following the confirmation of loss of pedal reflex, cervical dislocation was manually conducted by applying firm pressure at the base of the skull while pulling the tail to ensure quick separation of the cervical vertebrae^35^.

#### Preparation of the extract for antidiabetic activity

The *A. monosperma* DAME extract was weighed at 100, 200, and 300 mg and placed in a mortar. Subsequently, one drop of Tween 80 was added until complete dissolution. Subsequently, distilled water was added gradually to achieve the desired concentrations (100, 200, and 300 mg%). Subsequently, the extract was filtered and administered to the rats at weight-based doses.

#### Preliminary oral toxicity evaluation

A preliminary oral toxicity screening was performed to assess the safety of DAME in fasted male albino rats (200–250 g). The study involved three groups (*n* = 6). DAME was given *via* oral gavage at doses of 100, 200, and 300 mg/kg body weight following an overnight fast. The three tested doses (100, 200, and 300 mg/kg b.w.) were selected because they fall within the range reported in the literature for pharmacologically active, non-lethal doses of Artemisia extracts in rodents according to Moharram et al., 2021^36^. Rats were monitored for 24 h for behavioral changes, such as tremors, convulsions, and incoordination, as well as respiratory issues like rapid or labored breathing. The dose levels were selected to provide an early indication of tolerability at relevant pharmacological doses, rather than to establish an LD₅₀ value.

#### Evaluating the antihyperglycemic effects of *A. monosperma* DAME

After overnight fasting, male albino rats (200–250 g; *n* = 6) were classified into five groups. The first group received the vehicle only (control). The second, third, and fourth groups were administered DAME extract by oral gavage at doses of 100, 200, and 300 mg, respectively. The fifth group received Diamicron^®^ (gliclazide) as the reference drug at a dose of 80 mg. Thirty min later, rats were orally loaded with sucrose (10 g/kg) dissolved in distilled water. This high-dose challenge was selected to induce a pronounced glycemic peak and better evaluate the extract’s anti-hyperglycemic potential, following previously validated protocols^37,38^. BGLs were measured at 0 (before the sucrose load) and at 30, 60, 90, and 120 min. after the sucrose load using a glucometer (Gluco Dr Super Sensor, All Medicus Co., Ltd., Anyang, Gyeonggi, Korea) to calculate the glucose area under the curve (AUC)^39^. Diamicron→ (Gliclazide, 80 mg) was used as the reference drug.

#### Induction of type-2 diabetes (T2D) in rats

The potential antidiabetic effect of DAME was tested in diabetic rats made hyperglycemic by a 4-week high-fat diet (HFD) containing 60% fat, 20% protein, and 20% carbohydrate, with a total energy of 25.07 KJ/g. After overnight fasting, the rats were injected *I.p.* with 35 mg/kg streptozotocin, which was prepared in 0.05 M cold citrate buffer at pH 4.5. Blood glucose levels were measured with a glucometer after 2 days, and rats with BGL ≥ 200 mg/dL were included in the experiment^40^.

Diabetic rats were randomly assigned to four groups (*n* = 6), with all groups fasted overnight before the first group received an oral vehicle (T2D control). The other three groups received diamicron as a standard antidiabetic drug (80 mg/kg) or DAME orally at specified doses (200 and 300 mg). After 1 h, each rat received an oral glucose dose of 1.0 g/kg. Blood glucose levels were then recorded at baseline (before glucose administration) and at 30, 60, 90, and 120 min. post-glucose load, using a glucometer.

### Anti-inflammatory effect of *A. monosperma* DAME

#### RAW 264.7 culture and viability assay

RAW 264.7 murine macrophages (ATCC, Manassas, VA, USA) were maintained in Dulbecco’s Modified Eagle Medium (DMEM; Invitrogen/Life Technologies) supplemented with 10% fetal bovine serum (Hyclone, GE Healthcare, USA), insulin at a final concentration of 10 µg/mL, and 1% penicillin–streptomycin (Sigma-Aldrich, St. Louis, MO, USA). Cells were incubated at 37 °C in a humidified environment containing 5% CO₂. RAW cell viability was assessed using the MTT-based in vitro toxicology assay kit (Sigma-Aldrich, USA). Cells were seeded at 1.2–1.8 × 104 cells per well and incubated at 37 °C in a CO_2_-enriched incubator with different concentrations of DAME (250, 63, 16, 4, and 1.0 µg/mL) for 24 h. After treatment, MTT solution was added to each well and incubated at 37 °C for 4 h. The optical density at 590 nm was measured using a microplate reader (Sunrise, TECAN Inc., USA) to assess cell viability, and the results were compared with those of untreated control cells.

#### Evaluation of inflammatory markers (TNF-α and CRP) by qRT-PCR

RAW264.7 cells were plated at a density of 1 × 10^6^ cells/well in 6-well plates and allowed to reach confluence. They were then treated with DAME at a concentration equivalent to 0.25 of the IC_50_ for two h, followed by the addition of 1 µg/mL LPS to induce inflammation. 24 h later, the cells were harvested for RNA extraction using a Qiagen RNA extraction kit (Sigma-Aldrich in Saint Louis, Missouri, USA). Reverse transcription was performed using a RevertAid™ H Minus Reverse Transcriptase kit (Sigma-Aldrich in Saint Louis, Missouri, USA), and mRNA levels of CRP and TNF-α were quantified using BioRad Sybr green PCR MMX with specific RT primers as provided in Table 1. qRT-PCR was conducted *via* a Rotor-Gene Q instrument (QIAGEN Hilden, Germany), and fold change was calculated using the 2^−ΔΔCt^ method. Beta-actin (ACTB) was used as a housekeeping gene. All experiments were done in triplicate to ensure accuracy.

**Table 1:** Primers for CRP, TNF-α, and the housekeeping gene ACTB.

TNF-α	F 5’- GGTGCCTATGTCTCAGCCTCTT-3’
	R 5’- GCCATAGAACTGATGAGAGGGAG-3’
CRP	F 5’- TCGTGGAGTTCTGGGTAGATGG-3’
	R 5’- TTCCCACCGAAGGAATCCTGCT-3’
ACTB	F 5’- CATTGCTGACAGGATGCAGAAGG-3’
	R 5’-TGCTGGAAGGTGGACAGTGAGG-3’

### Evaluation of wound healing

#### BJ cell culture and treatment

Human foreskin fibroblast (BJ) cells (CRL-2522; ATCC) were cultured in Dulbecco’s Modified Eagle Medium (Gibco; Thermo Fisher Scientific, Inc.) supplemented with 10% fetal bovine serum (Sigma-Aldrich-Merck KGaA; Darmstadt, Germany) and 1% penicillin-streptomycin (Beijing Solarbio Science & Technology Co., Ltd.). The cells were maintained in a cell incubator (Thermo Fisher Scientific, Inc.) at 37 °C with 5% CO_2_ and full humidity. When BJ cells reached the logarithmic growth phase, they were seeded into 6-well plates at a density of 2 × 10^5^ cells per well and incubated for 24 h. The wells were divided into control (untreated) cells and *A. monosperma* aerial parts DAME-treated cells.

#### Wound-healing assay

The migration of BJ cells was evaluated using a wound-healing assay. Cells from each group were prepared at a concentration of 3 × 10^5^/mL and seeded into 6-well plates to form a cell monolayer. A scratch was created in the cell layer using a 10-µL pipette tip, and debris was removed. Images were captured at 0 and 24 h post-scratching with an inverted microscope (Olympus Corp.), and the wound-healing rate was quantified using ImageJ (version 1.46r; National Institutes of Health).

### Assessments of antioxidant activity

#### DPPH radical scavenging activity

The procedure followed the protocol of Sharma et al. (2009)^41^. *A. Monosperma* aerial parts DAME was applied in concentration series (12.5, 25, 50, 100, and 200) µg/mL. Ascorbic acid served as the reference, and DMSO as the negative control. Each measurement was done in triplicate. Nonlinear regression analysis was performed to determine the IC_50_, the concentration required to inhibit free radical formation by 50%.

#### Superoxide radical inhibition activity (O_2_^-^.)

The assay followed the standard procedure outlined by Xiang & Ning (2008). *A. Monosperma* aerial parts DAME at concentrations (12.5, 25, 50, 100, and 200 µg/mL) were tested. Each test was performed in triplicate. Ascorbic acid served as the reference, while DMSO was the negative control. The IC_50_ value was determined by nonlinear regression analysis.

#### Nitric oxide (NO) scavenging activity

It was evaluated using the methods outlined by^42^. Ascorbic acid served as the reference, and DMSO as the negative control. Absorbance was measured at 546 nm in triplicate. The IC_50_ value was obtained from the dose-response curve using non-linear regression analysis.

### Statistical analysis

All results are expressed as Mean ± Standard Deviation (SD). Data was analyzed using One-way Analysis of Variance (ANOVA). Significant differences between the mean values of the treatment groups and the control groups were determined using Tukey’s post-hoc multiple comparison test. A *p*-value of less than 0.05 was considered statistically significant. Statistical analysis was performed using GraphPad Prism software version 9.0 (La Jolla, CA, USA).

### In silico molecular docking

The structures of inducible nitric oxide synthase (iNOS), particularly its catalytic oxygenase domain, and the sulfonylurea receptor 1 (SUR1) with PDB ID of 3E7G^43^ and 6PZA^44^, respectively, were downloaded from the Protein Data Bank^45^. Both proteins were purified and prepared for docking using AutoDock Tools 1.5.7^46^. The structures of compounds **1–25** were minimized and prepared with Open Babel^47^ in PyRx^48^. A grid box measuring 30 × 30 × 30 Å, centered at 55, 17, and 83, was set up for screening against iNOS. For SUR1, a grid box of the same size, centered at 203, 282, and 219, was used. The parameters for each protein were first validated by docking its co-crystallized ligand using AutoDock Vina^49^ through PyRx, and by calculating the root mean square deviation (RMSD). Compounds **1–25** were screened against each target using consistent parameters. The results were analyzed using Maestro 12.8^50^.

## Results

The 2-DPC of DAME revealed a mixture of polyphenolic compounds, as evidenced by its behavior under UV light and upon spraying with Naturstoff and FeCl_3_ reagents.

### HPLC/HRMS analysis for DAME of *A. monosperma* aerial part

**Fig. 1:**
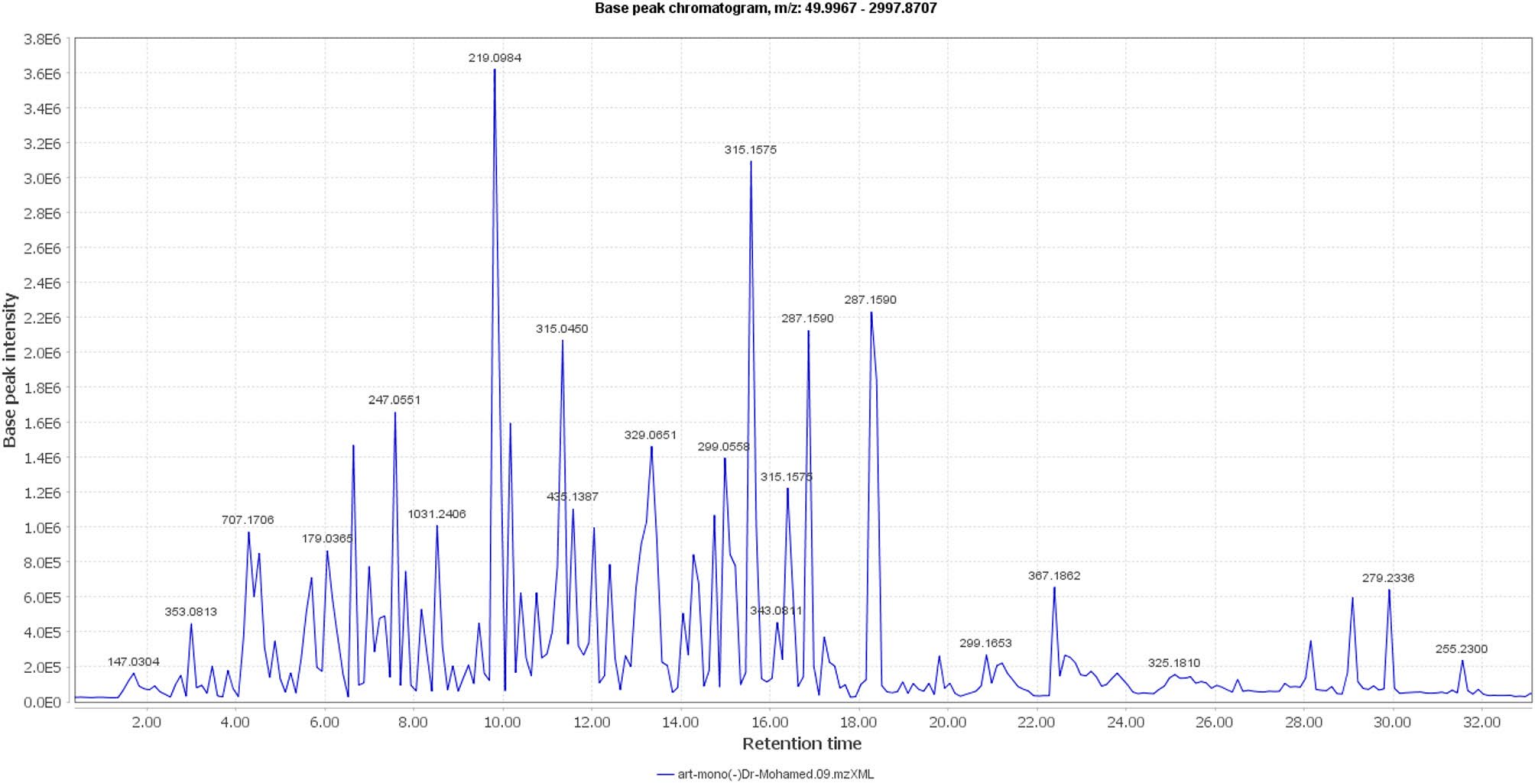
Total negative ionization mode HRHPLC/MS chromatogram of the aerial parts of *A*. *monosperma* DAME.

The HRHPLC/MS analysis of the DAME of *A*. *monosperma* tentatively identified 25 secondary metabolites in the negative mode, including phenolic acid derivatives, flavonoids, and terpenes (Table 2; Fig. 1).

**Table 2:** Tentative identification of metabolites in *A. monosperma* DAME extract by HPLC–HRMS based on accurate mass and literature comparison.

No	Compound	*Rt	[M-H]^-^	Theoretical [M–H]⁻	Δppm	Exact mass	MF	References
**1**	Caffeoylquinic acid isomer	2.99	353.0813	353.0878	18.4	354.0951	C_16_H_18_O_9_	^24^
**2**	Protocatechuic acid	3.46	153.0172	153.0184	7.8	154.0266	C_7_H_6_O_4_	^54^
**3**	Adipostatin E	3.82	333.0669	333.2666	−	334.2872	C_22_H_38_O_2_	^84^
**4**	Sesamin	4.52	353.0813	353.0878	18.4	354.1103	C_20_H_18_O_6_	^64^
**5**	Caftaric acid	5.70	311.0721	311.0403	-	312.0481	C_13_H_12_O_9_	^51^
**6**	Caffeic acid	6.05	179.0365	179.0346	10.6	180.0423	C_9_H_8_O_4_	^28,53^
**7**	Dihydroartemisinic acid	6.64	235.0951	235.1695	−	236.1776	C_15_H_24_O_2_	^85^
**8**	Vanillic acid sulfate	7.58	247.0551	247.0004	−	247.9991	C_8_H_8_O_7_S	^86^
**9**	Dicaffeoylquinic acid	7.81	515.1099	515.1190	17.7	516.1268	C_25_H_24_O_12_	^24,51^
**10**	Dihydroartemisinic aldehyde	9.81	219.0984	219.1852	−	220.1827	C_15_H_24_O	^85^
**11**	Caffeoyl feruloylquinic acid	10.75	529.1336	529.1357	4.0	530.1424	C_26_H_26_O_12_	^55^
**12**	Isorhamnetin	11.34	315.0450	315.0506	17.8	316.0583	C_16_H_12_O_7_	^27^
**13**	Malaferin B	11.57	435.1387	435.1296	20.9	436.1158	C_24_H_20_O_8_	^87^
**14**	Chicoric acid hexoside isomer	12.04	635.1391	635.1517	19.8	636.1326	C_28_H_28_O_17_	^88^
**15**	Taxifolin	12.40	303.1619	303.0503	−	304.0583	C_15_H_12_O_7_	^60^
**16**	Tricin	13.34	329.0651	329.0452	−	330.0740	C_17_H_14_O_7_	^66^
**17**	Rosmarinic acid	14.04	359.0784	359.0779	1.4	360.0845	C_18_H_16_O_8_	^53^
**18**	Methylcatechin	14.28	303.1619	289.0718	-	304.0947	C_16_H_16_O_6_	^53^
**19**	Methoxy apigenin	14.98	299.0558	299.0553	1.7	300.0634	C_16_H_12_O_6_	^18,59^
**20**	Protocatechuic acid-*O*-hexoside	15.57	315.0450	315.0506	17.8	316.0794	C_13_H_16_O_9_	^54^
**21**	Eriodictyol	16.86	287.1590	287.0553	−	288.0634	C_15_H_12_O_6_	^61^
**22**	Feruoylquinic acid	22.39	367.1862	367.1034	−	368.1107	C_17_H_20_O_9_	^55^
**23**	Artecanin	28.15	277.2169	277.1130	−	278.1154	C_15_H_18_O_5_	^63^
**24**	*p*-coumaroyl ester	29.91	279.2336	279.0452	−	280.0583	C_13_H_12_O_7_	^56^
**25**	Pinocembrin	31.56	255.2300	255.0661	−	256.0736	C_15_H_12_O_4_	^62^

#### Phenolic acids

Phenolic acids are commonly found throughout the genus Artemisia^51^. Based on their chemical scaffold, phenolic acids are sub-classified into hydroxybenzoic and hydroxycinnamic acid derivatives^52^. In the current study, we identified three hydroxybenzoic acid derivatives (**2**, **8**, **20**) and nine cinnamic acid derivatives (**1**,** 5**,** 6**,** 9**,** 11**,** 14**,** 17**,** 22**, and **24**). Compounds **2**, **8**, and **20** were identified as protocatechuic acid, vanillic acid 4-sulfate, and protocatechuic acid -*O*-hexoside with *m/z* [M-H]^−^ 153.0172, 247.0551, and 315.0450, respectively. Compound **6** was identified as caffeic with *m/z* [M-H]^−^ 179.0365, while **1**,** 5**,** 9**,** 11**,** 14**,** 17** and **22** are caffeic acid derivatives; they were identified as caffeoylquinic acid isomer **1**, caffeoltartaric acid **5** (caftaric acid), dicaffeoylquinic acid **9**, caffeoylferuloylquinic acid **11**, dicaffeoyl- tartaric acid hexoside **14** (chicoric acid hexoside isomer), rosmarinic acid **17** and 5-feruoylquinic acid **22** with *m/z* [M-H]^−^ 353.0813, 311.0721, 515.1099, 529.1336, 636.512, 359.0784 and 367.1862, respectively. Moreover, one coumaric acid derivative, *p*-coumaroyl ester **24**, with *m/z* [M-H]^−^ 279.2336 was also identified. Compounds **1**, **6**, **9**, **17**, and **24** were previously identified from *A*. *monosperma* leaves^24,28,51,53^, while compounds **2**, **5**, **11**, **20**, and **22** were identified from the genus Artemisia before^51,53–56^. However, this is the first time these compounds have been identified from *A*. *monosperma*, along with compounds **4** and **18**, which are reported here for the first time from the genus.

#### Flavonoids

Flavonoids are commonly found in the genus Artemisia^57,58^. They include flavonol derivatives such as isorhamnetin, **12**, and dihydroquercetin (taxifolin) **15**, with *m/z* [M-H]^−^ 315.0450 and 303.1619, respectively; flavone derivatives like tricin, **16** (*m/z* [M-H]^−^ 329.0651), and methoxy apigenin **19** [M-H]^−^ 299.0558; flavanones such as eriodictyol **21** [M-H]^−^ 287.1590 and pinocembrin, **25** [M-H]^−^ 255.230; as well as flavan-3-ols like malaferin B, **13** and methylcatechin **18**, with m/z [M-H]^−^ 435.1387 and 303.1619, respectively. It was reported that compounds **12**, **18**, and **19** had previously been identified from *A*. *monosperma*^18,26,27,53,59^, while compounds **15**^60^, **21**^61^, and **25**^62^ were previously identified in the genus, but for the first time from *A*. *monosperma*.

Additionally, compounds **13** and **16** were identified for the first time from both the plant and genus.

#### Miscellaneous

Three sesquiterpenes are identified: dihydroartemisinic acid,**7** ([M-H]^−^ 235.0951); dihydroartemisinic aldehyde, **10** ([M-H]^−^ 219.0984); and artecanin, **23** ([M-H]^−^ 277.2169), a resorcinol derivative; adipostatin, **3** ([M-H]^−^ 333.06690); and one lignan derivative, sesamin, **4** ([M-H]^−^ 353.0813). Compounds **7** and **10** were previously identified from *A*. *monosperma* (El Naggar, 2012), while compounds **4** and **23** were previously reported from the genus^63,64^ and are reported here for the first time from the plant, along with compound **3**.

#### Anti-diabetic potential of *A. monosperma* DAME

#### Preliminary oral toxicity evaluation

No adverse effects were observed at the tested doses (100, 200, and 300 mg/kg) under the conditions of this study. Previous studies have shown low toxicity in certain Artemisia extracts. we cite specific research on an Artemisia extract made with a similar 80% aqueous methanol extract^36^, which more closely resembles the current DAME.

#### Estimation of the antihyperglycemic activity

The antihyperglycemic potential of *A. monosperma* DAME was evaluated using the SLM. As shown in Table 3, the DAME extract exhibited a dose-dependent reduction in the glucose area under the curve (AUC). While the lower doses showed a modest decrease, the 300 mg/kg dose significantly reduced the AUC by 19% compared with the SLM control group, demonstrating efficacy comparable to the reference drug, Diamicron^®^.

**Table 3:** Screening of *A. monosperma* DAME oral administration in normal rats on blood glucose levels using SLM.

Sample	conc	AUC (mg/min/dL)	% Reduction in BGL compared to control
DAME of *A. monosperma*	100 mg%	193.63 ± 16.12^*^	13.90
	200 mg%	192.08 ± 21.94^*^	14.58
	300 mg%	182.75 ± 17.81^**^	18.73
Diamicron^®^	80 mg	189.21 ± 10.45^**^	15.86

Data is presented as mean AUC ± SD. * Significant (*P* < 0.05), ** (*P* < 0.01) or *** (*P* < 0.001) compared to control group using One-way ANOVA followed by Tukey’s post-hoc test. SLM: sucrose load model. *N* = 6.

#### Influence of DAME of *A. monosperma* on blood glucose levels in T2D rats

A T2D animal model was used to investigate the antihyperglycemic effects of *A. Monosperma* aerial parts DAME after oral administration of the two selected doses, 200 and 300 mg%, compared with diamicon→. The findings indicated that 300 mg% significantly improved glucose tolerance, resulting in approximately a 26% decrease in blood glucose levels, an effect comparable to diamiconⓇ (26.9%) (Table 4).

**Table 4:** The antidiabetic effect of *A. monosperma* DAME by an OSTT in T2D rats.

Sample	conc	AUC (mg/min/dL)	% Reduction in BGL
DAME of *A. monosperma*	200 mg%	756.63 ± 63.35	03.34
	300 mg%	578.54 ± 20.13^***^	26.09
Diamicron^®^	80 mg	572.15 ± 14.61^***^	26.91

Data is shown as mean AUC ± SD. ***: *P* < 0.001 compared to the control group using One-way ANOVA followed by Tukey’s post-hoc test. OSTT: oral sucrose tolerance test. (*n* = 6).

### The anti-inflammatory effect of *A. Monosperma* DAME

**Fig. 2:**
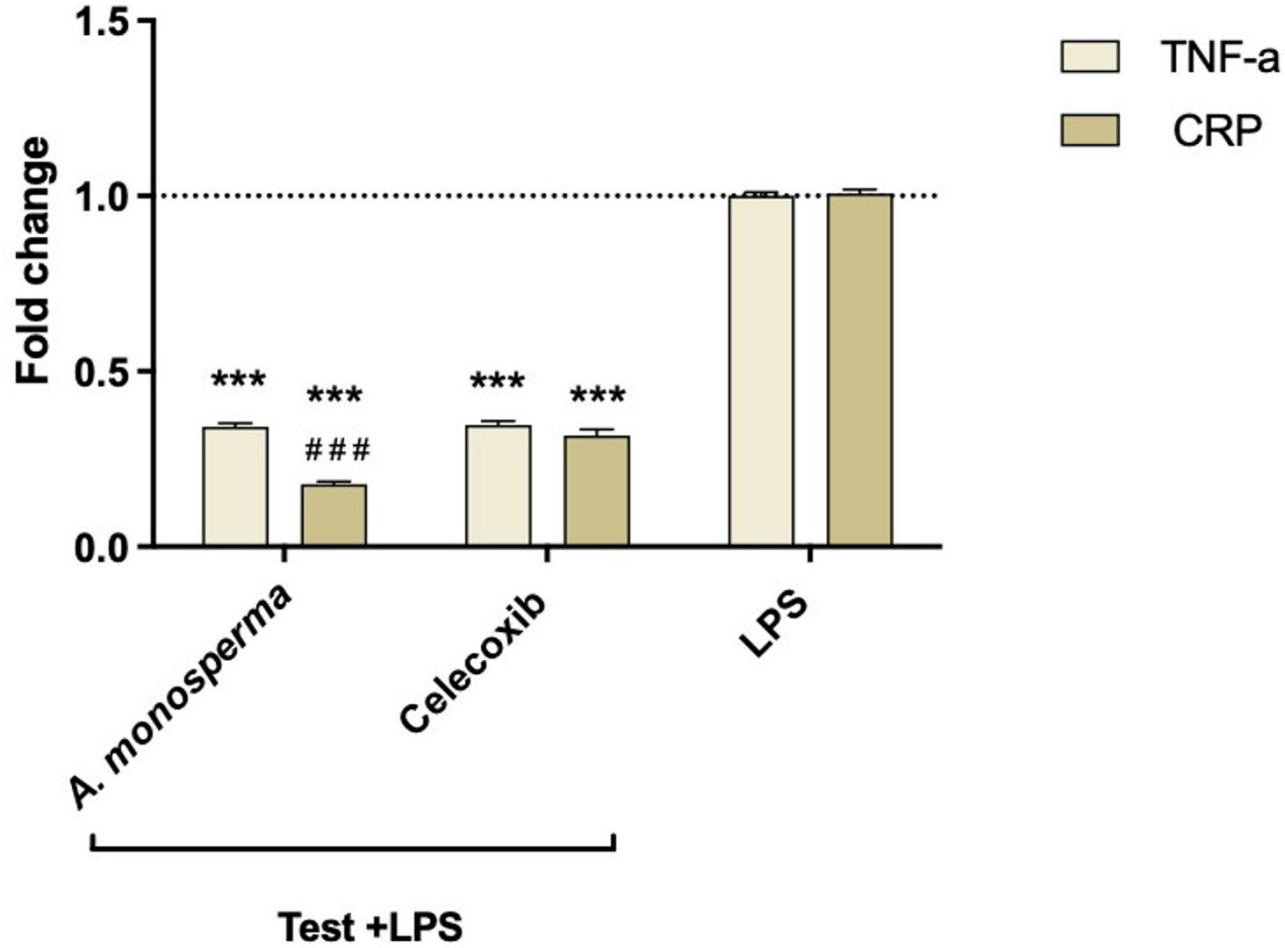
Anti-inflammatory effect of *A. monosperma* DAME in LPS-treated Raw cells, ^***^: *P* < 0.001 vs. LPS control, and ^# # #^: *P* < 0.001 vs. celecoxib *via* one-way ANOVA, followed by post hoc test. Data are expressed as the mean ± SD of three experiments.

To evaluate the anti-inflammatory effects, we measured changes in TNF-α and CRP gene expression in RAW264.7 cells following LPS stimulation. DAME exhibited significant anti-inflammatory activity (*p* < 0.001), with reductions of approximately 66% in TNF-α and 82% in CRP expression compared to LPS-treated control (Fig. 2). Additionally, DAME exerted a greater inhibitory effect on CRP expression (*p* < 0.001) than celecoxib, which reduced CRP expression by 70%.

### Effect of *A. monosperma* DAME on wound healing

**Fig. 3:**
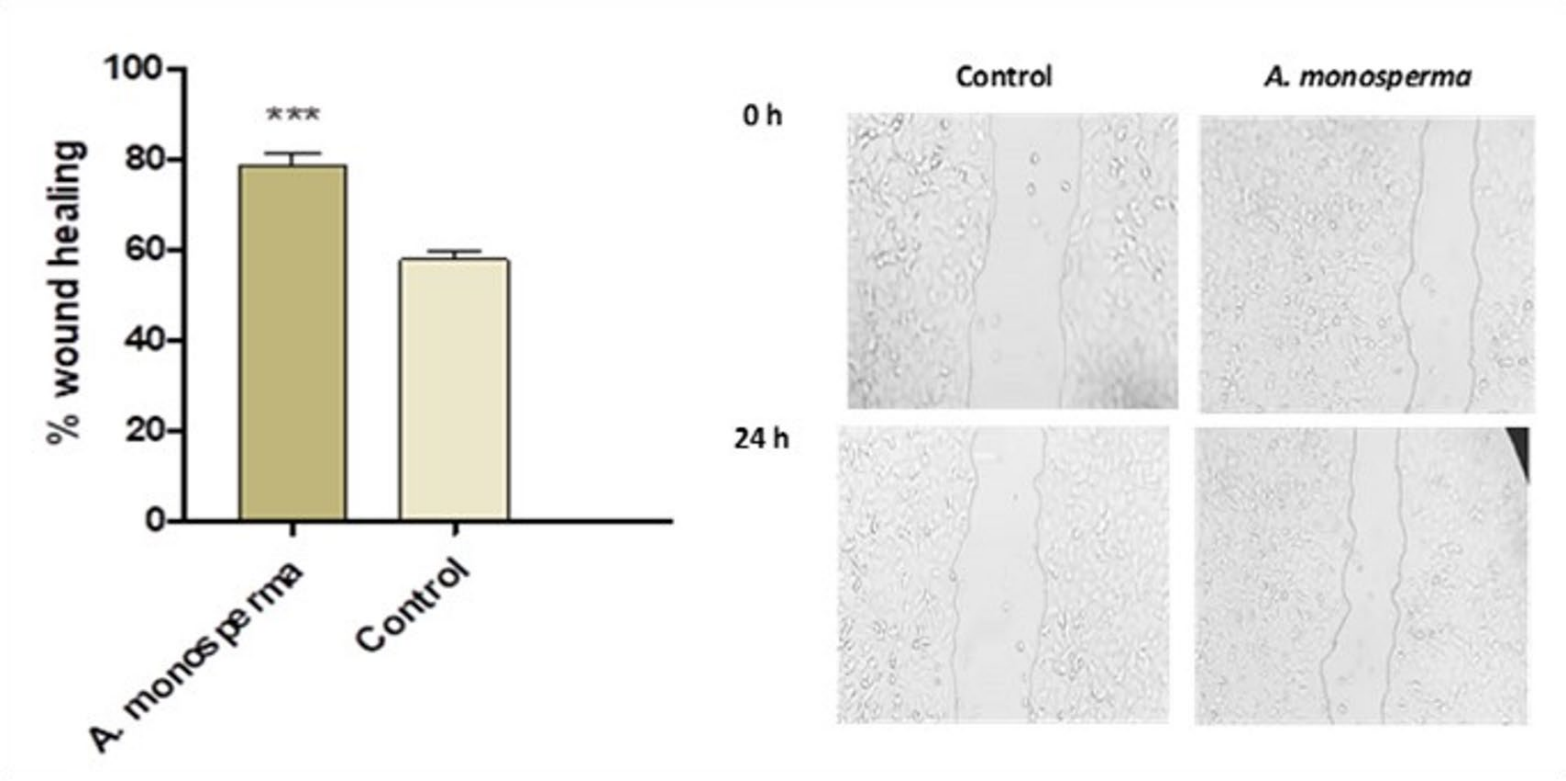
Effect of *A. monosperma* DAME *on* wound healing *via* BJ cells’ migration assay ^***^: *P* < 0.001 vs. control cell via unpaired t test. Data are expressed as the mean ± SD of three experiments.

The results of the wound-healing assay are shown in Fig. 3. Wound healing in *A. Monosperma-*treated BJ cells reached approximately 79% after 24 h, whereas control, untreated BJ cells reached about 58% after 24 h. Wound healing rate in case of DAME was significantly higher compared to control cells (*p* < 0.001).

### The antioxidant activity of *A. monosperma* aerial parts DAME

The DAME of *A. monosperma* showed promising antioxidant activity compared to ascorbic acid. As shown in Table 5, the superoxide scavenging activity of DAME (IC_50_ = 11.05 µg/mL) demonstrated superior effects compared to ascorbic acid. Additionally, DAME exhibited good DPPH and NO scavenging activity (IC_50_ = 34.66 and 56.12 µg/mL, respectively) relative to ascorbic acid (Table 5).

**Table 5:** Evaluation of antioxidant activity of *A. monosperma* DAME.

Samples	IC_50_ (µg/mL)		
	DPPH	Superoxide radical	Nitric oxide
DAME	34.66	11.05	56.12
Ascorbic acid	19.65 ± 0.39	21.6 ± 0.43	37.91

Data is shown as mean ± SD.

### In silico docking study

The binding energies of the compounds screened in silico for their affinity to both iNOS and SUR1 are listed in Table 6. For iNOS, the energies ranged from − 10.2 to −6.3 kcal/mol, compared to −8.9 kcal/mol for the co-crystallized ligand (PDB ID: AT2, or ethyl 4-[(4-methylpyridin-2-yl) amino] piperidine-1-carboxylate). Similarly, for SUR1, energies ranged from − 11.0 to −6.1 kcal/mol, versus − 10.4 kcal/mol for the co-crystallized drug (glibenclamide). The RMSD values for each docked co-crystallized ligand, AT2 for iNOS and glibenclamide for SUR1, relative to their native structures were 0.511 and 1.512 Å, respectively.

**Table 6:** Virtual screening results against iNOS and SUR1.

Ligand number	Binding energy (kcal/mol)		Ligand number	Ligand number	
	iNOS	SUR1		iNOS	SUR1
**1**	−9.3	−8.6	**14**	−10.0	−9.8
**2**	−6.3	−6.1	**15**	−9.0	−9.5
**3**	−8.0	−7.3	**16**	−7.7	−8.8
**4**	−9.8	−11.0	**17**	−10.1	−8.4
**5**	−8.7	−8.1	**18**	−8.0	−8.8
**6**	−7.3	−6.8	**19**	−8.6	−9.4
**7**	−7.1	−8.2	**20**	−8.4	−8.0
**8**	−7.4	−7.0	**21**	−9.3	−9.4
**9**	−10.2	−10.3	**22**	−9.7	−8.8
**10**	−7.2	−7.9	**23**	−7.6	−8.0
**11**	−10.2	−9.8	**24**	−8.4	−7.7
**12**	−8.2	−9.5	**25**	−8.9	−9.1
**13**	−9.7	−9.7	**Co-crystallized** ^***a***^	−8.9	−10.4

^*a*^Co-crystallized ligand: AT2 for iNOS or glibenclamide for SUR1.

**Fig. 4:**
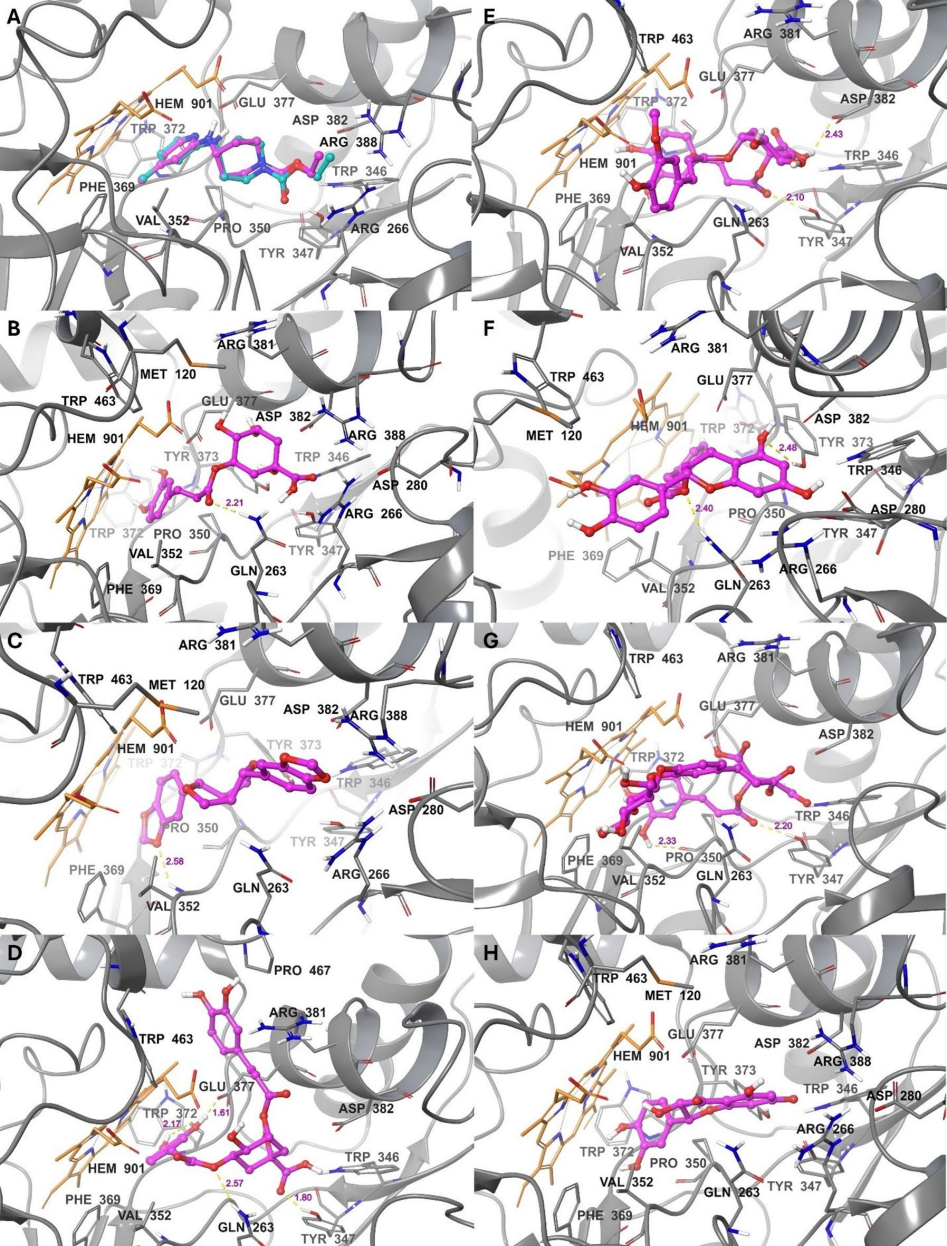
Binding modes to iNOS: (**A**) Co-crystallized (teal) vs. docked (magenta) AT2. Compounds (**B**) **1** (Caffeoylquinic acid isomer), (**C**) **4** (sesamin), (**D**) **9** (dicaffeoylquinic acid), (**E**) **11** (caffeoylferuloylquinic acid), (**F**) **13** (malaferin B), (**G**) **14** (chicoric acid hexoside isomer), and (**H**) **15** (taxifolin). Ligands are displayed as ball-and-stick models, while residues and heme (HEM901) are shown as tubes. Hydrogen bonds are depicted as yellow dashed lines with their lengths in magenta.

**Fig. 5:**
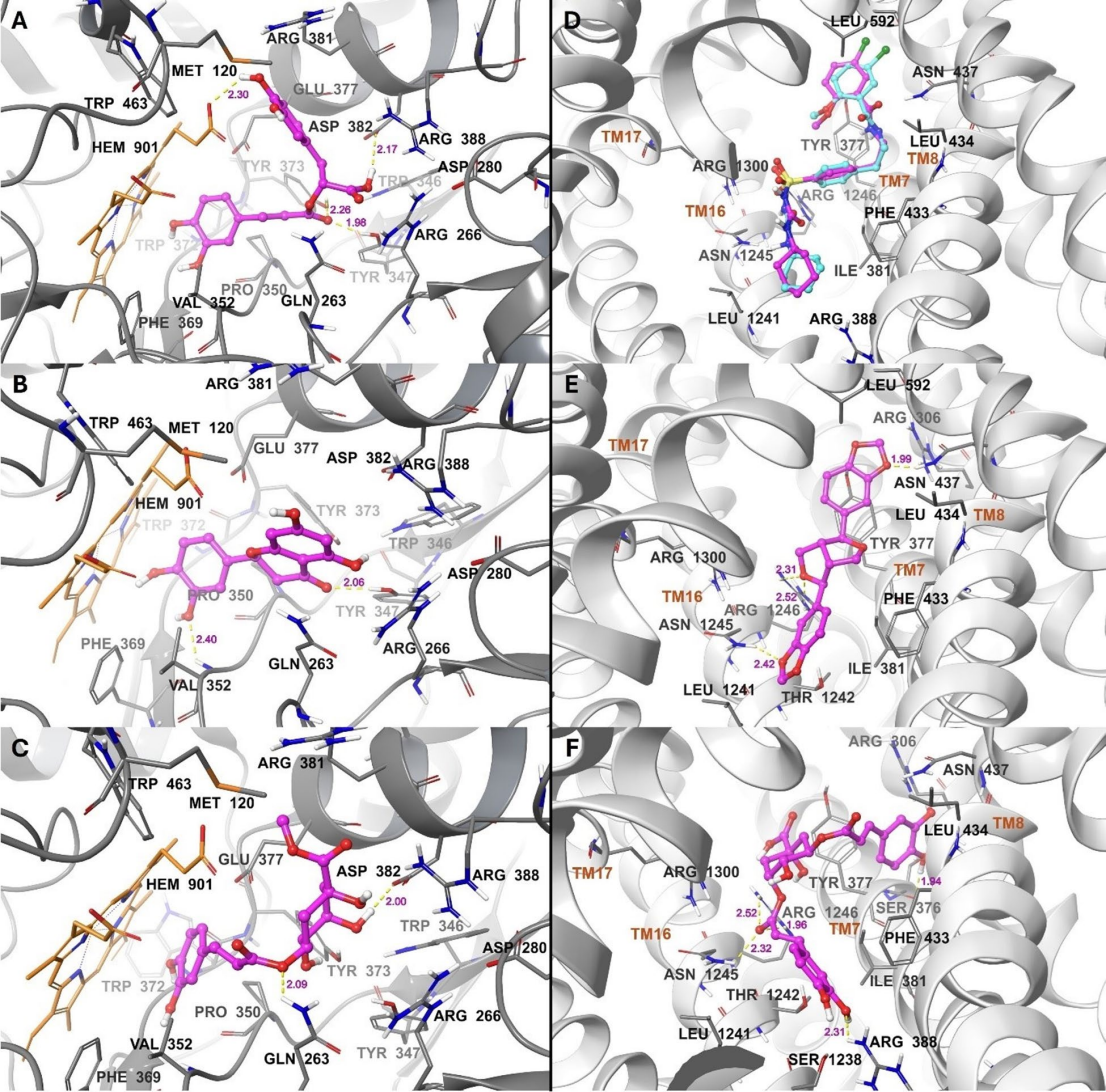
Binding modes to iNOS *(continued)*: compounds (**A**) **17** (rosmarinic acid), (**B**) **21** (eriodyctiol), and (**C**) **22** (methylchlorogenate). Binding modes to SUR1: (**D**) Co-crystallized (teal) vs. docked (magenta) glibenclamide. Compounds (**E**) 4 (sesamin) and (**F**) **9** (dicaffeoylquinic acid). Ligands are displayed as balls and sticks, while residues and heme (HEM901) are shown as tubes. Hydrogen bonds are depicted as yellow dashed lines with their lengths in magenta.

Ten compounds showed better binding affinities to iNOS than the co-crystallized ligand (i.e., with binding energies ≤ −9.0 kcal/mol), including compounds **9** and **11** (−10.2), **17** (−10.1), **14** (−10.0), **4** (−9.8), **13** and **22** (−9.7), **1** and **21** (−9.3), and **15** (−9.0). Fourteen other compounds possessed energy values ranging from − 8.9 to − 7.0 kcal/mol: compounds **25** (−8.9), **5** (−8.7), **19** (−8.6), **20** and **24** (−8.4), **12** (−8.2), **3** and **18** (−8.0), **16** (−7.7), **23** (−7.6), **8** (−7.4), **6** (−7.3), **10** (−7.2), and **7** (−7.1). The highest binding energy observed was − 6.3 kcal/mol for compound **2**. The docking validation and binding modes of the top compounds with energies ≤ −9.0 kcal/mol for iNOS are shown in Table 7; Fig. 4A–H, and 5A–C.

**Table 7:** Binding interactions of top compounds to iNOS.

Ligand number	Hydrogen bonds		Interacting moieties	
	Count	Length (Å)	Ligand	Residue
**1**	1	2.21	Caffeoyl C = O	GLN263 NH_2_
**4**	1	2.58	Benzodioxole O	VAL352 NH (backbone)
**9**	4	2.57 1.80 2.17 1.61	Propenoate O Quinic acid COOH Caffeoyl OH Caffeoyl OH	GLN263 NH_2_ TYR347 OH TRP372 C = O (backbone) GLU377 COO^−^
**11**	2	2.10 2.43	Caffeoyl C = O Quinic acid COOH	TYR347 OH ASP382 COO^−^
**13**	2	2.40 2.48	Propenoate O Dihydroxychromane OH	GLN263 NH_2_ TYR373 OH
**14**	2	2.20 2.33	Caffeoyl C = O Caffeoyl OH	TYR347 OH PRO350 C = O (backbone)
**15**	Nil	–	–	–
**17**	4	1.98 2.26 2.17 2.30	Caffeoyl C = O Caffeoyl C = O Propanoic COOH Catechol OH	TYR347 OH TYR373 OH ASP382 COO^−^ Heme COO^−^
**21**	2	2.06 2.40	Dihydroxychromanone C = O Catechol OH	TYR347 OH VAL352 NH (backbone)
**22**	2	2.09 2.00	Propenoate O Quinic acid OH	GLN263 NH_2_ ASP382 COO^−^

**Fig. 6:**
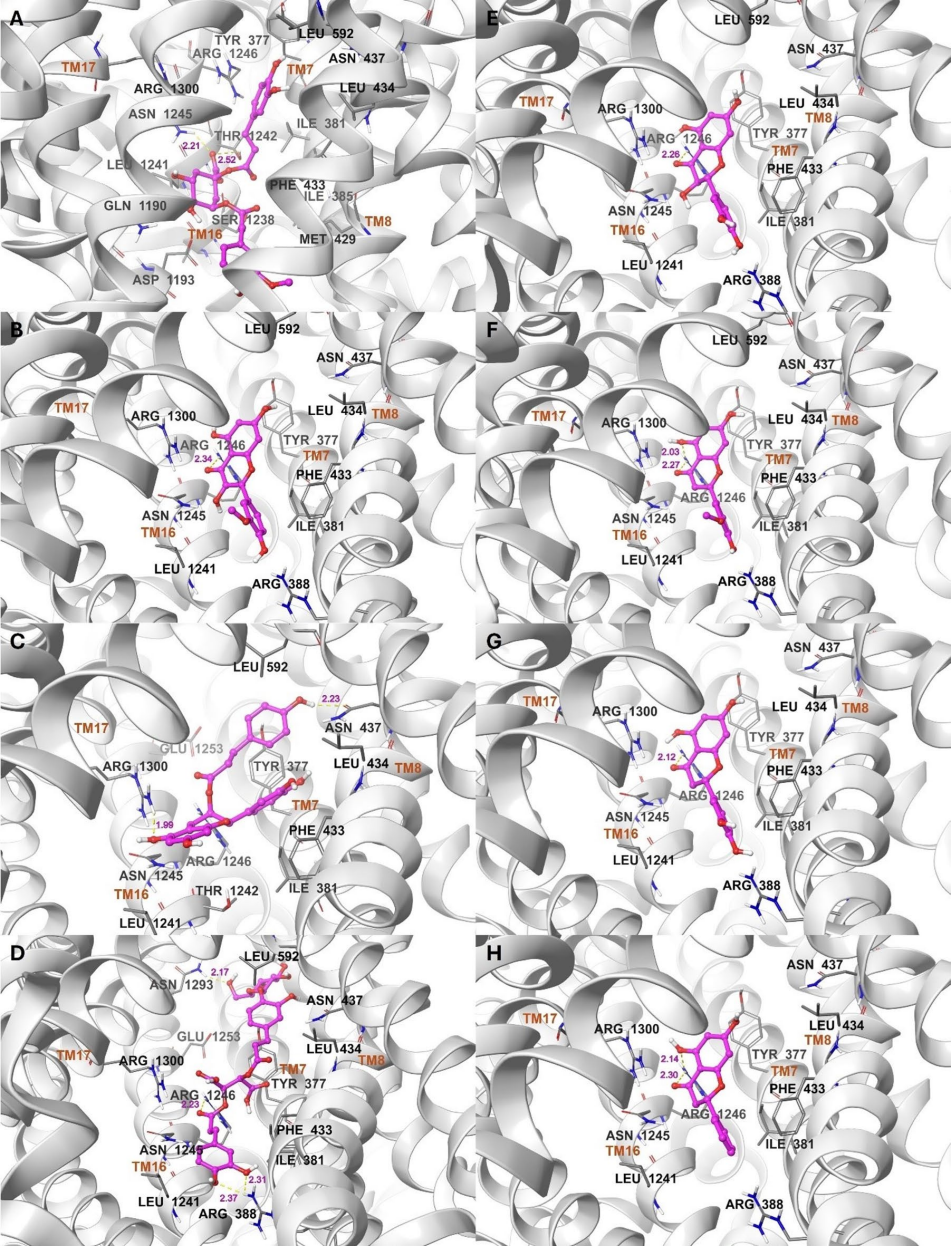
Binding modes to SUR1 *(continued)*: Compounds (**A**) **11** (caffeoylferuloylquinic acid), (**B**) **12** (isorhamnetin), (**C**) **13** (malaferin B), (**D**) **14** (chicoric acid hexoside isomer), (**E**) **15** (taxifolin), (**F**) 19 (methoxyapigenin), (**G**) **21** (eriodyctiol), and (**H**) **25** (pinocembrin). Ligands are displayed as balls and sticks, while residues are shown as tubes. Hydrogen bonds are depicted as yellow dashed lines with their lengths in magenta.

To SUR1, compound **4** showed a lower binding energy of −11.0 kcal/mol than that of the co-crystallized ligand. Additionally, compound **9** exhibited an energy of −10.3 kcal/mol, similar to that of glibenclamide. Eight other compounds had energy scores of ≤ −9.0 kcal/mol, including compounds **11** and **14** (−9.8), **13** (−9.7), **12** and **15** (−9.5), **19** and **21** (−9.4), and **25** (−9.1). Thirteen compounds displayed energy values ranging from − 8.9 to −7.0 kcal/mol: compounds **16**, **18**, and **22** (−8.8), **1** (−8.6), **17** (−8.4), **7** (−8.2), **5** (−8.1), **20** and **23** (−8.0), **10** (−7.9), **24** (−7.7), **3** (−7.3), and **8** (−7.0). Compounds **6** and **2** recorded the highest scores of −6.8 and − 6.1 kcal/mol, respectively. The docking validation and binding modes of the top compounds with energies of ≤ −9.0 kcal/mol to SUR1 are shown in Table 8; Fig. 6D–F, and 6A–H.

**Table 8:** Binding interactions of top compounds to SUR1.

Ligand number	Hydrogen bonds		Interacting moieties	
	Count	Length (Å)	Ligand	Residue
**4**	4	1.99 2.42 2.31/2.52	Benzodioxole O Benzodioxole O Hexahydrofurofuran O	ASN437 NH_2_ ASN1245 NH_2_ ARG1246 NH_2_^+^/NH_2_
**9**	5	1.91 2.31 2.32 2.52/1.96	Caffeoyl OH Caffeoyl OH Caffeoyl C = O Caffeoyl C = O	SER376 C = O (backbone) ARG388 NH_2_^+^ ASN1245 NH_2_ ARG1246 NH_2_^+^/NH_2_
**11**	2	2.52 2.21	Quinic acid COOH Quinic acid COOH	THR1242 OH ASN1245 NH_2_
**12**	1	2.34	Trihydroxychromone C = O	ARG1246 NH_2_^+^
**13**	2	2.23 1.99	Hydroxyphenyl OH Dihydroxychromane OH	ASN437 C = O ARG1300 NH_2_
**14**	4	2.31 2.37 2.23 2.17	Caffeoyl OH Caffeoyl OH Caffeoyl C = O Hexose CH_2_OH	ARG388 NH_2_^+^ ARG388 NH_2_^+^ ARG1246 NH_2_^+^ ASN1293 NH_2_
**15**	1	2.26	Trihydroxychromanone C = O	ARG1246 NH_2_^+^
**19**	2	2.27 2.03	Dihydroxychromone C = O Dihydroxychromone OH	ARG1246 NH_2_^+^ ARG1246 NH_2_^+^
**21**	1	2.12	Dihydroxychromanone C = O	ARG1246 NH_2_^+^
**25**	2	2.30 2.14	Dihydroxychromanone C = O Dihydroxychromanone OH	ARG1246 NH_2_^+^ ARG1246 NH_2_^+^

## Discussion

Medicinal plants are often used to prevent and treat human diseases due to their phenolic compounds^65^. Many studies have shown that plants with antihyperglycemic effects act through multiple mechanisms, including improving insulin sensitivity in target cells, increasing insulin secretion, and promoting β-cell regeneration in the pancreatic islets of Langerhans^66^. Several researchers have identified flavonoids and phenols as major contributors to their antidiabetic effects^67^.

In this context, this study evaluated the antidiabetic, anti-inflammatory, and antioxidant potential of *A*. *monosperma* DAME.

The anti-inflammatory activity of DAME from *A. monosperma* was proven by a reduction in TNF-α and CRP expression in response to LPS stimulation of RAW macrophages. This effect may be linked to the presence of hydroxycinnamic acids^68^.

The anti-inflammatory activity of DAME may reduce inflammation-driven insulin resistance and enhance blood glucose regulation by modulating macromolecular signaling. Furthermore, the anti-inflammatory properties of DAME, along with its demonstrated ability to promote wound healing, may improve delayed wound-healing problems in diabetes^69^. The DAME is rich in phenolic compounds, such as hydroxycinnamic acids and flavonoids, which possess significant anti-inflammatory activity^70,71^.

Enhancing antioxidant capacity is a key therapeutic approach for managing diabetic complications related to oxidative stress. Due to their phenolic compounds, which are associated with enhanced antioxidant capacity, the interest in medicinal plants has grown^72^. DAME’s antioxidant capacity underscores its potential to exert antioxidant and antidiabetic effects by reducing oxidative stress and/or enhancing the antioxidant system. Based on phenolic content, the extract exhibits potential reducing power and scavenging activity, which correlate with its promising antioxidant capacity, as demonstrated herein by DAME’s ability to scavenge DPPH, superoxide, and NO radicals^72^.

Phenolic compounds, such as hydroxycinnamic acids and flavonoids, can reduce free radicals, including superoxide anions, which are ordinary free radicals increased during oxidative stress, originating from auto-oxidation or enzymatic reactions leading to additional oxidative damage^73^; by boosting antioxidant enzyme activity, thereby increasing antioxidant values, as seen in this study^68^. *A*. *monosperma* DAME might strengthen the antioxidant system in diabetes by directly neutralizing free radicals and may also help lower hyperglycemia by improving insulin sensitivity and reducing *β*-cell damage.

Nitric Oxide (NO) is a key biological mediator and a free radical generated under physiological conditions by almost all mammalian cells. It concerns the regulation of the cardiovascular and immune systems in mammals. Excess NO levels are associated with several diseases^42^.

The nitric oxide synthase (NOS) enzyme family produces NO by converting L-arginine into L-citrulline and NO *via* oxidation reactions. These enzymes consist mainly of two domains: An N-terminal oxygenase domain and a C-terminal reductase domain, both connected by a calcium-calmodulin binding region^74,75^. There are three main isoforms of NOS: neuronal, inducible, and endothelial, among which iNOS is induced by inflammatory cytokines or pathogens to produce large amounts of NO to combat inflammation or infection^74–77^. NO acts as a defense molecule against infectious microorganisms and regulates the development, activity, and death of various inflammatory and immune cells^78^. However, overexpression or dysregulation of iNOS results in excessively high NO levels, which are linked to multiple diseases such as inflammatory conditions, rheumatoid arthritis, and diabetes. Therefore, controlling the expression and activity of iNOS is essential for maintaining its beneficial functions and preventing its harmful effects^43,76,77^. Thus, all compounds **1–25** were virtually screened against the iNOS active site within the oxygenase domain, which contains the substrate-binding site and the heme cofactor, to help identify potential inhibitors. The compounds fit into the active site, including the main residues GLN263, ARG266, TYR347, PRO350, and ARG388, as well as heme. While docking the co-crystallized ligand (AT2) achieved an energy of −8.9 kcal/mol (Table 6; Fig. 4A), ten compounds had much better binding affinities to iNOS. According to their chemical classification, the top compounds are cinnamic acids **9**,** 11**,** 17**,** 14**,** 22**, and **1**; lignan **4**; catechin **13**; flavanone **21**; and flavonol **15**. Within the energy range from − 8.9 to −7.0 kcal/mol, there were fourteen ligands, including cinnamic acids **5**, **6**, and **24**, catechin **18**, flavonone **25**, flavonol **12**, flavones **16** and **19**, phenolic acids **8** and **20**, sesquiterpenes **7**, **10**, and **23**, alongside resorcinol **3**. The phenolic acid **2** ranked last in binding to iNOS, with a binding energy of −6.3 kcal/mol (Table 6). Consequently, among compounds **1–25**, the identified cinnamic acids and lignans appear to be the most promising as iNOS inhibitors, followed by the catechins, flavanols, flavonones, and flavones. The identified resorcinol, sesquiterpenes, and phenolic acids could be the least promising. The interactions of the top ten ligands with iNOS are detailed in Table 7; Figs. 4B–H, and 5A–C. These primarily involved hydrogen bonding and π-π stacking. Compound **1** formed a hydrogen bond between its caffeoyl C = O and GLN263 (Fig. 4B), while Compound **4** formed a hydrogen bond between its benzodioxole oxygen and VAL352 (Fig. 4C). Compound **9** showed four hydrogen bonds: one between the propenoate oxygen and GLN263, another between quinic acid COOH and TYR347, and two involving caffeoyl OH groups with TRP372 and GLU377 (Fig. 4D). Compounds **1**, **4**, and **9** also participated in π-stacking with the heme via the caffeoyl ring in compounds **1** and **9**, and through the benzodioxole ring in compound **4**. The caffeoyl C = O and quinic acid COOH of compound **11** formed hydrogen bonds with TYR347 and ASP382, respectively. Also, its aromatic rings were π-stacked with heme (Fig. 4E). Compound **13** formed hydrogen bonds with GLN263 and TYR373 via its propenoate oxygen and dihydroxychromane OH, respectively, and its hydroxyphenyl group π-stacked with heme (Fig. 4F). Compound **14** had its caffeoyl C = O and OH groups hydrogen-bonded to TYR347 and PRO350, respectively (Fig. 4G). Compound **15** engaged in π-stacking with heme through its catechol moiety (Fig. 4H). Compound **17** interacted through its caffeoyl C = O and propanoic COOH with TYR347, TYR373, and ASP382, and via its catechol OH with heme (Fig. 5A). The dihydroxychromanone C = O and catechol OH of compound **21** were bound to TYR347 and VAL352 (Fig. 5B). Compound **22** exhibited hydrogen bonds with GLN263 and ASP382 *via* its propenoate oxygen and quinic acid OH, respectively, and π-stacked with heme through its caffeoyl ring (Fig. 5C). According to previous reports, caffeoylquinic acid isomer (**1**)^79^ was reported for its potential as an iNOS inhibitor through in-vitro and in-silico study showing binding affinity that coincides with our reported results. Ejembi et al. (2021)^80^ reported the binding affinity of several phenolic derivatives identified from *A*. *annua*, including caffeoylquinic acid and di-*O-*caffeoylquinic acid, as iNOS inhibitors, and their results also showed a binding affinity score that matched ours^80^.

In pancreatic β-cells, the ATP-sensitive potassium (KATP) channel regulates insulin secretion based on blood glucose levels. The channel consists of four pore-forming subunits constituting the inward rectifying potassium channel (Kir6.2), surrounded by four regulatory sulfonylurea receptor 1 (SUR1) subunits^1,81^. SUR1 is part of the ATP-binding cassette (ABC) transporter superfamily, which transports different molecules across cell membranes^44,82^. Each SUR1 contains two transmembrane domains (TMD1 and TMD2), two cytoplasmic nucleotide-binding domains (NBD1 and NBD2), and an N-terminal transmembrane domain (TMD)^81,83^. Sulfonylureas and glinides are two types of insulin secretagogues used to treat type 2 diabetes. They bind to a specific pocket between the transmembrane (TM) α-helices of TMD1 and TMD2 in SUR1, reducing insulin secretion and lowering high blood glucose levels^44,82^. Compounds **1**–**25** were also in silico screened against the binding site of sulfonylureas and glinides to assess their potential as SUR1 inhibitors. A receptor structure in complex with glibenclamide, a sulfonylurea analog of gliclazide used in the antidiabetic screening, was selected. Docking the co-crystallized glibenclamide yielded a binding energy of −10.4 kcal/mol (Table 6; Fig. 5D). The identified lignan **4** showed stronger binding to SUR1, with an energy of −11.0 kcal/mol. Additionally, the cinnamic acid **9** obtained a comparable binding energy of −10.3 kcal/mol to that of glibenclamide. Moreover, eight other ligands achieved energy scores of ≤ −9.0 kcal/mol. Based on their classification, these top ten compounds include lignan **4**, cinnamic acids **9**, **11**, and **14**, flavonols **12** and **15**, flavanones **21** and **25**, flavone **19**, and catechin **13**. Among the remaining ligands, thirteen attained energy scores between − 8.9 and − 7.0 kcal/mol, involving cinnamic acids **1**, **5**, **17**, **22**, and **24**, sesquiterpenes **7**, **10**, and **23**, flavone **16**, catechin **18**, phenolic acids **8** and **20**, and resorcinol **3**. Cinnamic acid **6** and phenolic acid **2** exhibited the weakest binding to SUR1, with binding energies of −6.8 and − 6.1 kcal/mol, respectively (Table 6). Thus, the identified lignans, flavonols, and flavonones are the most promising SUR1 inhibitors, followed by cinnamic acids, flavones, and catechins. The identified sesquiterpenes, phenolic acids, and resorcinol could be the least promising. Presented in Table 8; Figs. 5D–F, and 6A–H are the binding interactions of the top ten binding ligands to SUR1. The compounds accommodated the same binding site as sulfonylureas and glinides, located between the TMs of TMD1 and TMD2, particularly TM7 and TM8 of TMD1, and TM16 and TM17 of TMD2. Compound **4** possessed hydrogen bonds between two of its benzodioxole oxygens and ASN437 and ASN1245, and between one of its hexahydrofurofuran oxygens and ARG1246 (Fig. 5E). Compound **9** formed hydrogen bonds between two caffeoyl OH groups and SER376 and ARG388, and between a caffeoyl C = O and both ASN1245 and ARG1246 (Fig. 5F). Compound **11** hydrogen-bonded to both THR1242 and ASN1245 through its quinic acid COOH moiety (Fig. 6A). A single hydrogen bond was disclosed between the trihydroxychromone C = O of compound **12** and ARG1246 (Fig. 6B). Compound **13** formed two hydrogen bonds: one between the hydroxyphenyl OH and ASN437, and another between the dihydroxychromane OH and ARG1300 (Fig. 6C). Compound **14** exhibited hydrogen bonds between two caffeoyl OH groups and ARG388, the caffeoyl C = O and ARG1246, and the hexose CH_2_OH and ASN1293 (Fig. 6D). ARG1246 showed significant hydrogen bonding to the trihydroxychromanone C = O of compound **15** (Fig. 6E), both the dihydroxychromone C = O and OH of compound **19** (Fig. 6F), the dihydroxychromanone C = O of compound **21** (Fig. 6G), and both the dihydroxychromanone C = O and OH of compound **25** (Fig. 6H).

## Conclusions

The HPLC/HRMS of *A. monosperma* DAME revealed the presence of 25 metabolites, mainly phenolic derivatives. DAME produces antihyperglycemic activity and significantly improves glucose tolerance compared to the control. Additionally, it exhibits notable anti-inflammatory and antioxidant activities, and the wound-healing rate with DAME was significantly higher than that in the control group (*p* < 0.001). Molecular docking of compounds **1**–**25** showed their fitting into the heme-containing active site of iNOS and the sulfonylurea binding site of SUR1. Several compounds exhibited higher binding affinities than the co-crystallized ligand for each target, indicating they may act as potential inhibitors of iNOS or SUR1. Notably, lignan four and cinnamic acids **9**, **11**, and **14** achieved remarkable binding affinities for both iNOS and SUR1. *A*. *monosperma* is considered a potential bioactive agent for the treatment of diabetes and inflammation. Nonetheless, additional research is needed to confirm their safety in clinical settings. Furthermore, additional OECD-compliant acute and subchronic toxicity studies are necessary to fully characterize the safety profile of *A. monosperma* DAME.

## Data Availability

All data generated or analyzed during this study are included in this published article [and its supplementary information files].

## Abbreviations

T1DM: Type 1 diabetes mellitus
T2DM: Type 2 diabetes mellitus
DAME: Defatted aqueous methanol extract of the aerial parts
HRHPLC/MS: High-resolution high-performance liquid chromatography coupled with mass spectrometry
Q-TOF LC/MS: Quadrupole time-of-flight liquid chromatography/mass spectrometry
SLM: Sucrose-loaded model
BGL: Blood glucose level
HFD: High-fat diet
DMEM: Dulbecco’s modified eagle medium
MTT: 3-(4,5-dimethylthiazol-2-yl)-2,5-diphenyltetrazolium bromide
CRP: C-reactive protein
TNF-α: Tumor necrosis factor-alpha
qRT-PCR: Quantitative reverse transcription polymerase chain reaction
BJ cells: Human foreskin fibroblast cells
DPPH: 2,2-diphenyl-1-picrylhydrazyl
O_2_^−^.: Superoxide radical
DMSO: Dimethyl sulfoxide
IC_50_: Half-maximal inhibitory concentration
NO: Nitric oxide
TAC: Total antioxidant capacity
iNOS: Inducible nitric oxide synthase
SUR1: Sulfonylurea receptor 1
PDB: Protein Data Bank
RMSD: Root mean square deviation
LPS: Lipopolysaccharides
ROS: Reactive oxygen species
KATP: ATP-sensitive potassium
NBD: Cytoplasmic nucleotide-binding domain
TMD: N-terminal transmembrane domain
TM: Transmembrane
